# Traumatic Partial Avulsion of Pinna Reconstruction with Limberg Flap

**Published:** 2018-05

**Authors:** Aravind Menon, Alagesan G

**Affiliations:** Shanthi Nursing Home, Surandai, Tirunelveli, India

**Keywords:** Pinna, Avulsion, Limberg flap, Reconstruction

## Abstract

Traumatic injuries of ear range from simple lacerations to complex avulsions and crush injuries. The complicating factors involved are cartilage involvement, poor vascularity of the region and need for high cosmetic satisfaction. Various techniques have been described for reconstruction of earlobe after traumatic injuries. Here, we describe the reconstruction of a partially avulsed pinna using the versatile Limberg flap with superior cosmetic outcomes. This is probably the first case to be described in literature to utilizing the Limberg flap for reconstruction of a traumatic avulsion of pinna.

## INTRODUCTION

Traumatic injuries of pinna pose are a real challenge to the reconstructive surgeon. Its prime role in contributing to the facial symmetry, presence of cartilage, direct exposure to external forces makes it a vulnerable organ for traumatic injuries. From repair of simple lacerations to complex avulsions, various techniques have been described by authors to achieve a cosmetically and anatomically satisfactory end result. Here, we describe the successful reconstruction of a partially avulsed pinna using the versatile Limberg flap and the superior cosmetic results achieved. This is probably the first case in literature to describe the utility of Limberg flap in reconstruction of avulsion injuries of pinna.^1^^-^^3^

## CASE REPORT

**Fig. 1:**
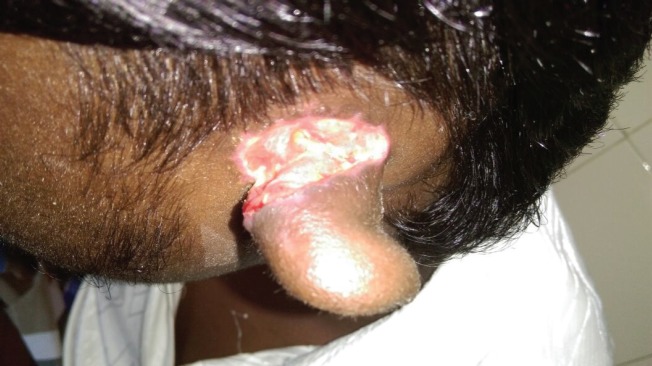
Infected raw area with slough.

**Fig. 2:**
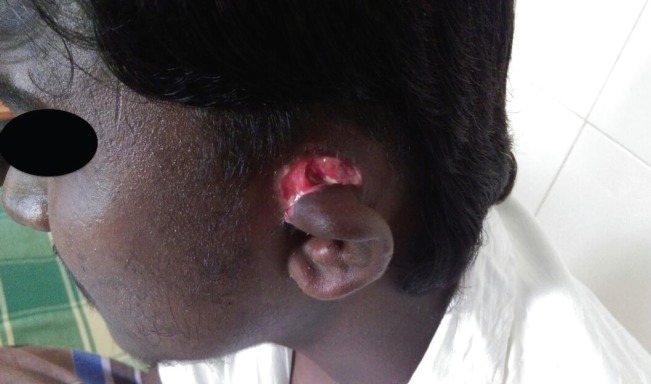
Granulated raw area after antibiotics and sloughectomy

A 19-year old male presented to the outpatient department (OPD) with history of sustaining injury to right pinna from a road traffic accident two weeks back. The patient sought treatment in a hospital where primary suturing was done but he developed wound site infection with foul smelling discharge and subsequently presented to us. Examination revealed partial avulsion of right pinna and separation of helix from post auricular skin with intervening sloughed out raw area (Figure 1). Patient was admitted and started on intravenous antibiotics and underwent a primary debridement and sloughectomy. Infection was progressively controlled with antibiotics and the raw area granulated (Figure 2). 

**Fig. 3:**
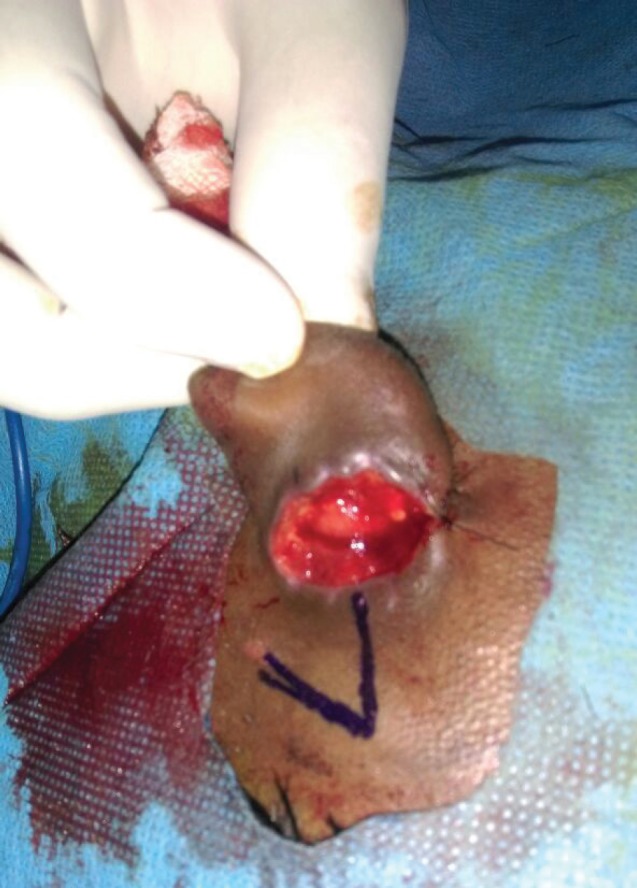
Raw area prepared into a rhomboid

**Fig. 4:**
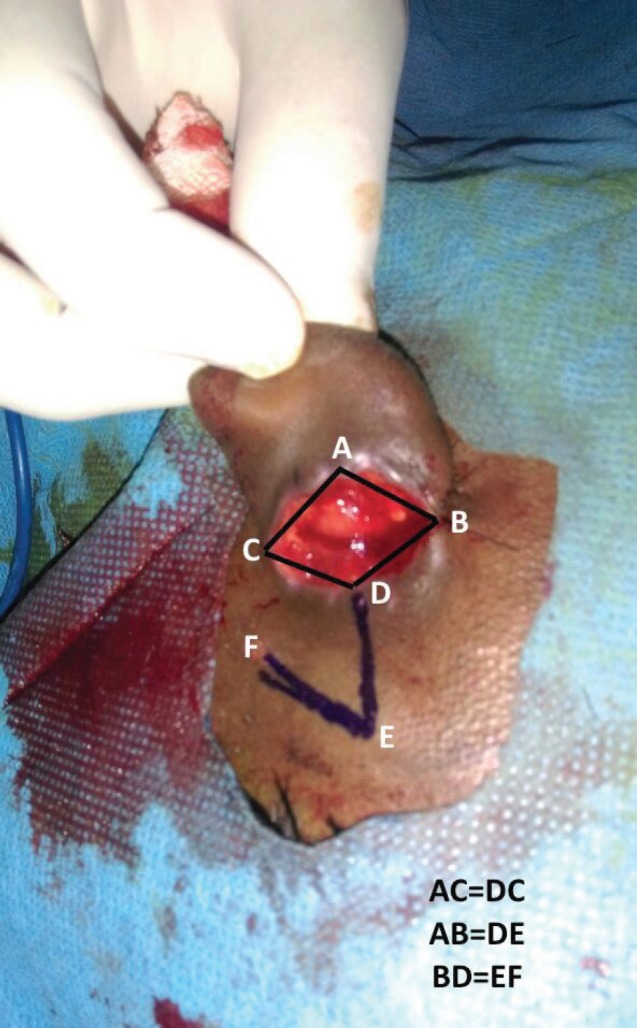
Rhomboid area depicted with markings.

**Fig. 5:**
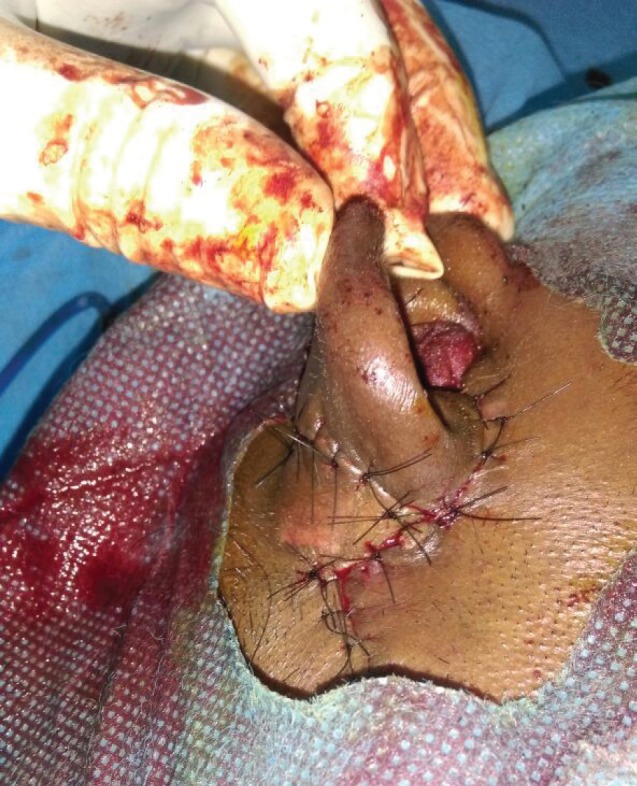
Post flap transposition.

**Fig. 6:**
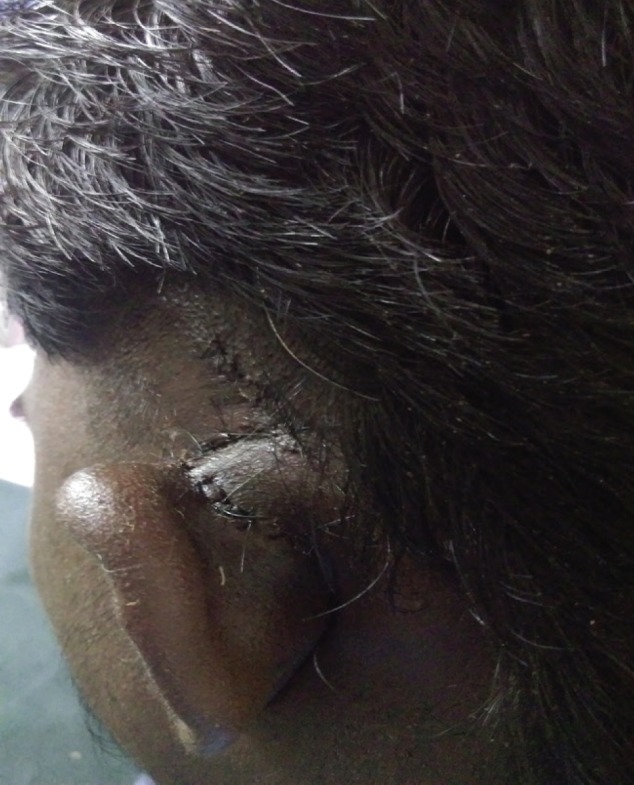
Healed wound after 1 week with good cosmesis

Due to recurrent risk of failure of sutures and for the benefit of cosmesis, it was planned to provide local flap cover for the raw area with Limberg transposition flap. The recipient area was prepared into a rhomboid as shown in (Figure 3 and 4) and the donor flap marked in the post auricular region as per measurements of the recipient area. Skin flap raised from the post auricular region was transposed to the recipient area and the donor flap sutured to complete the Limberg transposition flap (Figure 5). Paraffin dressing was applied. There was excellent wound healing as well as cosmesis since the scar was hidden in the post auricular region (Figure 6). 

## DISCUSSION

The traumatic external ear poses a challenge to the reconstructive surgeon. The protruded configuration and the presence of cartilage sandwiched between the skin makes it vulnerable to traumatic injuries and post traumatic infections. The usual mode of injuries are bite injuries, injuries in sportspersons, road traffic accidents and burns. When Kolodzynski *et al.* reviewed 105 patients who underwent auricular reconstruction post trauma, the commonest etiology was bite injury (22%) followed closely by traffic accidents (17%).^1^ Similar results were pointed out in the study by Steffen *et al. *where 35% of 74 patients who were a part of the study had bite injuries to pinna followed by traffic accidents which contributed 34%.^2^

Upper third injuries of pinna were common than any other part.^1^ The challenge to the reconstructive surgeon is to do an anatomical repair of the pinna as well as to provide acceptable cosmesis at the same time. Various techniques and methods have been described by authors highlighting the successful repair of traumatic injuries of pinna. Singh *et al.* described the use of doubled-over Limberg flap in reconstruction of ear lobule in 6 patients.^3^ Chattopadhyay *et al.* demonstrated the Gavello flap technique from post auricular mastoid region in ear lobule reconstruction in 3 patients.^4^ In the large volume study of 105 patients requiring post traumatic reconstruction by Kolodzynski *et al.*,^1^ apart from the use of costal cartilage for reconstruction in 53 patients, the skin cover for the pinna was provided using skin pockets in 53 patients, post auricular flap in 21 patients , tissue expansion in 12 and temporoparietal fascia flap in 12 patients. 

Kyrmizakis *et al.*^5^ described non microsurgical technique of pinna reconstruction in 2 cases of traumatic avulsion known as the Baudet technique which was first described by Baudet *et al.*^6^ in 1972. Park *et al.*,^7^ Destro and Speranzini^8^ independently described techniques for reconstruction of cartilage by sandwiching it between post auricular flap and facial artery flap. Manoli *et al.*^9^ described a retroauricular transposition flap as an innovative method of pinna reconstruction. Apart from these techniques, microsurgical reimplantation and repair of the pinna injuries are gaining popularity with successful case reports from numerous authors.

The versatile rhomboid flap was first described by Professor Limberg in 1928.^10^ It is a parallelogram with two angles of 120^0^ and two of 60^0^ which can be modified as per the size of the lesion. Limberg flap has been utilized for reconstructive techniques in various parts of body by various authors.^10^ However, its utility in reconstruction of pinna have been rarely described. Singh *et al.* described a technique of reconstruction of earlobe in 6 patients using modifications of Limberg flap.^3^ Ibrahim described a similar technique of reconstruction of ear lobule defect using Limberg flap.^11^

No literature was found describing the use of this flap in avulsion injuries of pinna. Thus, we believe this is the first case to be reported where the simple and versatile Limberg flap has been used to reconstruct a partially avulsed pinna as described. This is a simple technique with high cosmetic satisfaction and superior results in terms of wound healing as is evident from our case report. Traumatic injuries of pinna pose a challenge to the reconstructive surgeon in terms of achieving a good anatomical and cosmetic repair. Out of the non-microsurgical repair techniques, the Limberg flap is a simple and cosmetically acceptable technique that can be utilized for the reconstruction of an avulsed pinna.

## CONFLICT OF INTEREST

The authors declare no conflict of interest.
